# Advances in magnetic field approaches for non-invasive targeting neuromodulation

**DOI:** 10.3389/fnhum.2025.1489940

**Published:** 2025-04-28

**Authors:** Mozhgan Alipour, Maryam Abdolmaleki, Yaser Shabanpour, Alireza Zali, Farzad Ashrafi, Shabnam Nohesara, Behnam Hajipour-Verdom

**Affiliations:** ^1^Functional Neurosurgery Research Center, Research Institute of Functional Neurosurgery, Shohada Tajrish Comprehensive Neurosurgical Center of Excellence, Shahid Beheshti University of Medical Sciences, Tehran, Iran; ^2^School of Medicine, Shahid Beheshti University of Medical Sciences, Tehran, Iran; ^3^Department of Biophysics, Faculty of Biological Sciences, Tarbiat Modares University, Tehran, Iran; ^4^Department of Medicine (Biomedical Genetics), Boston University Chobanian and Avedisian School of Medicine, Boston, MA, United States; ^5^Department of Integrative Oncology, Breast Cancer Research Center, Motamed Cancer Institute, Academic Center for Education, Culture and Research (ACECR), Tehran, Iran

**Keywords:** neuromodulation, magnetic fields, magnetothermal stimulation, ion channels, deep brain stimulation

## Abstract

Neuromodulation, the targeted regulation of nerve activity, has emerged as a promising approach for treating various neurological and psychiatric disorders. While deep brain stimulation has shown efficacy, its invasive nature poses substantial risks, including surgical complications and high costs. In contrast, non-invasive neuromodulation techniques, particularly those utilizing magnetic fields (MFs), have gained increasing attention as safer, more accessible alternatives. Magnetothermal stimulation has emerged as an innovative method that enables precise modulation of neuronal ion channels through localized heating induced by interaction of MF with biological tissues. This review discusses the principles of MF-based neuromodulation and highlights the critical role of ion channels in synaptic transmission, and the therapeutic potential of these advanced techniques. Additionally, it highlights key challenges such as spatial targeting precision, safety considerations, and the long-term effects of magnetic exposure on brain function. The findings presente the promise of MF-based neuromodulation as a non-invasive, highly targeted therapeutic strategy for conditions such as epilepsy, movement disorders, and neurodegenerative diseases, with potential applications in chronic pain management and future clinical interventions.

## 1 Introduction

**FIGURE 1:**
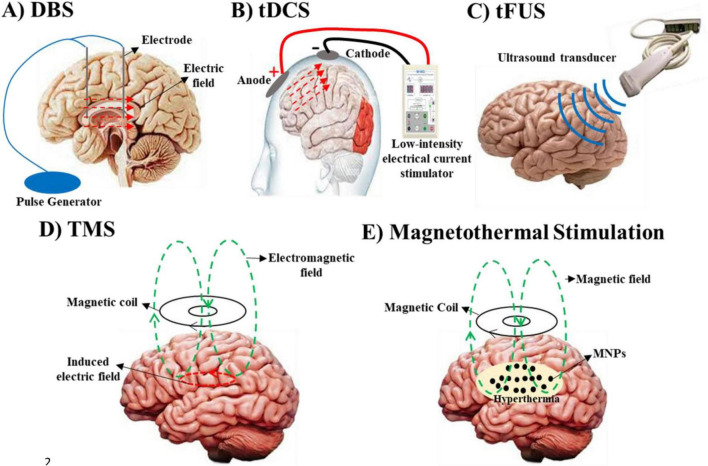
Various neuromodulation strategies, organized by their primary targets, clinical applications, and associated challenges. **(A)** Deep brain stimulation (DBS) modulates neuronal membrane potentials to treat Parkinson’s disease, essential tremor, and dystonia, though its invasive nature poses risks such as infection and bleeding. **(B)** Transcranial direct current stimulation (tDCS) similarly alters neuronal membrane potentials and is used to manage depression and cognitive impairments. However, it is limited by difficulties in precisely localizing target regions. **(C)** Transcranial focused ultrasound (tFUS) targets mechanical and pressure-sensitive ion channels for the treatment of Parkinson’s, essential tremor, and dystonia, but it carries the challenge of potential unintended effects on adjacent tissues. **(D)** Transcranial Magnetic Stimulation (TMS) modulates neuronal activity through the interaction of magnetic and electric fields. A rapidly changing magnetic field, generated by an electric current in a coil, induces an electric field in the brain tissue via electromagnetic induction. This electric field influences ion channel gating and neuronal membrane potentials via Lorentz forces acting on moving ions, enabling precise modulation of neural circuits. TMS is used to treat conditions such as epilepsy, Parkinson’s disease, schizophrenia, multiple sclerosis, chronic pain, depression, anxiety, and stroke. **(E)** Magnetothermal Stimulation uses magnetic hyperthermia to affect ion channels and neuronal excitability, showing promise for treating Parkinson’s, epilepsy and depression, while uncertainties regarding the biocompatibility and safety of magnetic nanoparticles (MNPs) remain.

Neuromodulation, the process of altering neural activity through targeted delivery of stimuli, has emerged as a transformative approach in treating neurological and psychiatric disorders (Luan et al., 2014). By modulating specific neural circuits, this technique offers precise control over brain function, providing alternatives to traditional pharmacological treatments, which are often associated with systemic side effects (Chen et al., 2024; Davis and Gaitanis, 2020). Over the past few decades, neuromodulation has evolved significantly, with techniques ranging from highly invasive methods, such as transcranial magnetic stimulation (TMS) and transcranial direct current stimulation (tDCS) (Marquez-Franco et al., 2022). While invasive methods such as DBS have shown remarkable efficacy in treating conditions such as Parkinson’s disease, the associated surgical risks-including infection and bleeding-have driven the development of safer and non-invasive alternatives (Jiang et al., 2020; Liu X. et al., 2022) (Figure 1).

Non-invasive neuromodulation techniques, such as TMS and tDCS, have revolutionized the field by enabling brain modulation without the need for surgical intervention (Alfihed et al., 2024). TMS uses MFs to induce electrochemical currents in targeted brain regions, while tDCS applies low-intensity electrical currents to modulate neuronal excitability (Ekhtiari et al., 2019). These methods have shown promise in treating conditions such as depression, anxiety, and epilepsy, offering greater accessibility and decreased risks compared to invasive procedures (Aderinto et al., 2024). However, their relatively diffuse stimulation patterns often result in limited spatial precision, potentially affecting unintended neural areas and compromising therapeutic outcomes. This limitation has driven the exploration of more targeted approaches, such as magnetothermal stimulation and focused ultrasound (FUS), which aim to achieve deeper and more precise modulation of neural activity (Liu X. et al., 2022; Woods et al., 2016).

Magnetothermal stimulation represents a cutting-edge advancement in non-invasive neuromodulation. By combining MFs with functionalized nanoparticles, this technique enables localized heating of targeted neural tissues, selectively modulating ion channels and neuronal activity with high precision (Kim et al., 2023). Similarly, FUS leverages acoustic waves to achieve deep brain modulation without the need for nanoparticles, offering an alternative pathway for precise neuromodulation. Both approaches address the critical need for greater specificity in non-invasive techniques, minimizing off-target effects and enhancing therapeutic efficacy (Jin et al., 2024).

This review provides the latest advancements in non-invasive neuromodulation, with a particular focus on MFs-based techniques. It explores the mechanisms, advantages, and challenges of established methods such as TMS and tDCS, as well as emerging approaches such as magnetothermal stimulation and FUS, highlighting a promising alternative for targeted neuromodulation. By integrating insights from neurobiophysics and biomedical engineering, this article presents a comprehensive overview of the evolving landscape of magnetic neuromodulation and its and its potential for clinical applications. Furthermore, it aims to advance the development of targeted, effective neuromodulation therapies and identify key directions for future research, paving the way for innovative therapeutic interventions in neurological and psychiatric disorders.

## 2 The potential of physics in neuroscience

The application of physical principles to the study and modulation of the nervous system establishes a foundational framework for advancing neuromodulation technologies. By exploring the interactions between electromagnetic fields (EMFs) and neuronal activity, this interdisciplinary field provides essential insights into how magnetic nanoparticle (MNP)-based approaches can achieve precise and targeted neuromodulation (Wang et al., 2023). Through its integration of physics and neuroscience-often referred to as neurobiophysics-this approach not only deepens our understanding of neural function but also drives the development of innovative therapies for neurological disorders (Alipour and Hajipour-Verdom, 2021; Pattabhi and Gautham, 2002).

A key strength of neurobiophysics is its emphasis on experimental control. Researchers meticulously design studies to isolate variables and observe neural processes under well-defined conditions, ensuring reproducibility and reliability. For instance, investigations into the effects of MFs on neuronal ion channels require precise control of environmental factors to confirm that observed changes are directly attributable to the applied stimuli. This rigorous approach is essential for advancing our understanding of how external physical forces, such as EMFs, can modulate neural activity (Goychuk, 2018; Mantovani, 2019).

The base of neurobiophysics is the use of physical laws to construct predictive models of neural phenomena. Drawing from principles of thermodynamics, electromagnetism, and quantum mechanics, these models provide a structured framework for interpreting complex neural processes. For example, electromagnetic theory-based models predict how specific frequencies and intensities of MFs interact with neural tissue, guiding the development of targeted neuromodulation therapies. Such models are invaluable for optimizing therapeutic outcomes while minimizing off-target effects (Takemura et al., 2020).

Neurobiophysicists also manipulate physical quantities-such as temperature, pressure, EMFs, sound, and electrical currents-to study their effects on biological systems (Alipour and Hajipour-Verdom, 2021). This approach enables precise control and measurement of neural activity, shedding light on how these parameters influence neuronal function at both microscopic and macroscopic levels. Techniques such as functional magnetic resonance imaging (fMRI), which measures brain activity by detecting changes in blood flow, and DBS, which modulates neural circuits through electrochemical impulses, are rooted in neurobiophysical principles (Yen et al., 2023). These applications highlight the field’s pivotal role in bridging theoretical insights with practical therapeutic tools (Chaari et al., 2011; Liu et al., 2019).

A fundamental assumption in neurobiophysics is that all neural activity can be explained through established physical laws (Franze and Guck, 2010). This premise drives research efforts to uncover mechanistic explanations for neural phenomena within the framework of known physics. Given the centrality of electrochemical signaling in nervous system function, many neurobiophysical models are grounded in electrical principles. These models elucidate the mechanisms underlying synaptic transmission, neural excitability, and the generation of complex neural rhythms, offering a quantitative understanding of brain function (Marshall et al., 2016; Sotero et al., 2024).

Neurobiophysics also plays a critical role in advancing MNP-based neuromodulation techniques. By examining how EMFs interact with neuronal structures, researchers can elucidate the mechanisms by which MNPs influence ion channel gating, membrane potentials, and synaptic activity (Chen et al., 2015). For instance, magnetothermal stimulation leverages localized heating and electromagnetic interactions to modulate neuronal excitability with high precision. This approach exemplifies the practical application of neurobiophysical principles, enabling targeted modulation of neural circuits while minimizing unintended effects on surrounding tissue (Munshi et al., 2017).

The insights gained from neurobiophysics have far-reaching implications for treating neurological disorders. By understanding the biophysical basis of ion channel function and neural excitability, researchers can develop more precise neuromodulation therapies for conditions such as epilepsy, chronic pain, and depression (Denison and Morrell, 2022). Additionally, neurobiophysics contributes to the design of advanced brain-machine interfaces, enhancing the integration of prosthetic devices with the nervous system. These advancements indicate the transformative potential of neurobiophysics in both basic research and clinical applications (Ebrahimzadeh et al., 2022; Yen et al., 2023).

In summary, neurobiophysics provides a mechanistic and quantitative foundation for understanding and modulating neural activity. By integrating physical principles with biological knowledge, this field not only advances our understanding of brain function but also drives the development of innovative neuromodulation techniques. From predictive modeling to the design of MNP-based therapies, neurobiophysics continues to shape the future of neuroscience and its applications in treating neurological disorders.

### 2.1 Basics of neuronal activity

Neurons rely on electrochemical processes to communicate, a fundamental mechanism that underpins everything from basic reflexes to complex cognitive functions. These processes are driven by the movement of ions across neuronal membranes, which is tightly regulated by ion channels (Gamper and Ooi, 2015). The flow of ions in response to various stimuli generates action potentials and synaptic signals, facilitating communication between neurons and other cells (Pastor and Attali, 2024; Spekker et al., 2023). Modulating ion channel activity provides a powerful means of influencing neuronal behavior, offering potential therapeutic avenues for a wide range of neurological and psychiatric disorders (Alipour et al., 2023; Chen et al., 2001). A deeper understanding of these principles is essential for exploring how MFs and other neuromodulation techniques can influence brain function, paving the way for innovative treatments and interventions.

Ca^2 +^ ions serve as vital second messengers, regulating key cellular processes such as neurotransmitter release and gene expression (Mitra et al., 2024). However, maintaining calcium homeostasis is essential, as its dysregulation can result in cellular toxicity and neuronal damage (Pikor et al., 2024). Pharmacological agents that target ion channels have been extensively employed to treat a range of medical conditions. For example, calcium channel blockers are particularly notable, they inhibit Ca^2 +^ influx, thereby reducing vascular smooth muscle contraction and cardiac muscle activity (Suzuki et al., 2024). This mechanism makes them highly effective in managing cardiovascular disorders, including hypertension, angina, and arrhythmias, as they lower blood pressure and improve blood flow by preventing excessive calcium-induced constriction of arterial walls (Arfat et al., 2023; Faucon et al., 2023). Additionally, during ischemic events, such as strokes, uncontrolled Ca^2 +^ influx exacerbates neuronal injury and cell death. To counteract these detrimental effects, cells rely on precise regulatory mechanisms to maintain balanced Ca^2 +^ levels, ensuring proper cellular function while minimizing the risk of toxicity (Nava et al., 2020; Ochoa et al., 2021; Qu et al., 2022; Singh et al., 2019).

Furthermore, potassium channel play a critical role in stabilizing neuronal membrane potentials, making them essential for proper cellular function. Blocking these channels can lead to membrane depolarization, increasing neuronal excitability and influencing physiological processes such as vascular smooth muscle contraction and neurotransmitter release (Jackson, 2017). Conversely, activating potassium channels hyperpolarizes neurons, reducing their likelihood of firing. This delicate balance highlights their importance as key regulators of neuronal activity and potential them as promising therapeutic targets for conditions such as depression and epilepsy (Sibille et al., 2015; Sun and Kapur, 2012).

Beyond the nervous system, potassium channels serve diverse roles in other tissues. In the heart, they contribute to the regulation of cardiac rhythm by stabilizing the membrane potential of cardiomyocytes (Grandi et al., 2017). In the brain, they modulate neuronal excitability and synaptic transmission, particularly in regions such as the hippocampus and cortex, which are crucial for memory and learning (Zhang J. et al., 2024). Additionally, in pancreatic β-cells, potassium channels respond to fluctuations in intracellular ATP levels, playing a pivotal role in regulating insulin secretion (Gil-Rivera et al., 2021). These varied functions indicate their importance in maintaining physiological homeostasis and their potential as targets for treating a wide range of disorders.

The gating process of channels is tightly regulated, ensuring that ion flow occurs only under precise conditions, which is essential for maintaining neuronal excitability and proper nervous system function (Alipour et al., 2023).

Ion channels can be categorized based on their gating mechanisms:

1. Voltage-gated ion channels that open or close in response to changes in the membrane potential. Voltage-gated Na^+^ and K^+^ channels are crucial for the initiation and propagation of action potentials, the electrochemical impulses that enable rapid signal transmission in neurons (Alipour et al., 2023; Gwanyanya and Mubagwa, 2022; Haustrate et al., 2019).
2. Mechanically-gated ion channels that respond to physical forces such as tension or pressure on the cell membrane. Found in sensory neurons, they play a key role in detecting touch, pressure, and stretch, contributing to our sense of touch and proprioception (Chakrabarti et al., 2024).
3. Ligand-gated ion channels that open in response to the binding of specific chemical messengers, such as neurotransmitters. They are essential for synaptic transmission, as they allow ions to flow in response to chemical signals released by neighboring neurons, facilitating communication within neural networks (Bowie, 2021).
4. Temperature-sensitive ion channels that open in response to changes in temperature, enabling neurons to detect heat or cold. They are integral to thermosensation, allowing organisms to respond to environmental temperature changes (Wang and Siemens, 2015).

Neurons exhibit the remarkable capability to detect and respond to both external and internal stimuli. Signal transmission initiates with the generation of an electrochemical impulse, which propagates rapidly along the axons. Upon reaching the axon terminal, the electrochemical signal triggers the release of neurotransmitters into the synaptic cleft, facilitating communication with adjacent neurons or target cells (Gamper and Ooi, 2015; Yang et al., 2022).

Synapses can be broadly classified into two types:

1. Electrical synapses that neurons are directly connected by gap junctions, which allow ions to pass freely between cells. This direct connection enables rapid and synchronized signal transmission, crucial for coordinating activity in regions such as the thalamus and hippocampus (Steyn-Ross et al., 2007).
2. Chemical synapses that involve a small gap, known as the synaptic cleft, between the presynaptic and postsynaptic neurons. When an action potential reaches the presynaptic neuron, it triggers the release of neurotransmitters, which diffuse across the synaptic cleft and bind to receptors on the postsynaptic neuron. This binding opens or closes ion channels, altering the postsynaptic membrane potential and propagating the signal (Thomson and Jovanovic, 2010; Toman et al., 2020).

### 2.2 Membrane potential and ion dynamics

The membrane potential of a neuron refers to the electrochemical potential difference across its membrane, which is crucial for neuronal excitability and signal transmission. At rest, the neuron maintains a stable baseline known as the resting membrane potential, typically around −70 mV. This potential is primarily sustained by the Na^+^/K^+^ pump, an active transport mechanism that expels Na^+^ from the cell and imports K^+^, creating an electrochemical gradient essential for neuronal function (Garg et al., 2022; Yoo et al., 2023).

The resting membrane potential can be quantitatively described using the Goldman-Hodgkin-Katz (GHK) equation (Equation 1) (Shirai et al., 2017; Sun, 2019), which accounts for the permeability of the membrane to different ions and their respective intracellular and extracellular concentrations.


(1) $$V_{m}=\frac{R\,T}{F}\,\ln\,\left(\frac{p_{k}\,{\left[K^{+}\right]}_{O}+p_{N\,a}\,{\left[N\,a^{+}\right]}_{O}+p_{C\,l}\,{\left[C\,l\right]}_{i}}{p_{k}\,{\left[K^{+}\right]}_{i}+p_{N\,a}\,{\left[N\,a^{+}\right]}_{i}+p_{C\,l}\,{\left[C\,l\right]}_{O}}\right)$$

Where:

1. V_m_ is the membrane potential,
2. R is the gas constant,
3. T is the temperature in Kelvin,
4. F is the Faraday constant,
5. P_k_, P_Na_, P_Cl_ represent the membrane permeability for potassium, sodium, and chloride ions, respectively,
x _o_ and [x]_i_ denote the extracellular and intracellular concentrations of the respective ions (K^+^, Na^+^ and Cl).

Once the membrane potential is determined, the current flowing across the neuronal membrane can be calculated using Ohm’s law (Equation 2). This equation relates the membrane current (I_m_) to the membrane conductance (g_m_) and the difference between the membrane potential (V_m_) and the equilibrium potential (E_m_):


(2) $$I_{m}=g_{m}\,\left(V_{m}-E_{m}\right)$$

This relationship is fundamental for understanding the dynamics of ion flow across the membrane, which directly influences neuronal signaling. The rate of ion flow, governed by the activity of ion channels, determines changes in membrane potential and, consequently, the initiation and propagation of action potentials. These processes are essential for neuronal communication, as they enable the transmission of electrochemical signals along neural networks.

## 3 The application of magnetic fields

MFs are generated by moving electric charges and can exert forces on other moving charges, such as the ions that flow through neuronal ion channels. When applied to biological tissue, MFs can induce electrochemical currents without direct physical contact, a phenomenon known as electromagnetic induction. This principle forms the foundation for using MFs in neuromodulation (Gorobets et al., 2024; Kohno et al., 2023). Through electromagnetic induction, time-varying MFs can generate electrochemical currents in targeted brain regions thereby influencing neuronal activity with precision. This mechanism supports techniques such as DBS, where magnetic pulses modulate brain activity by altering neuronal excitability (Kaur et al., 2019; Shibata et al., 2022). Additionally, MFs can impact the behavior of ion channels, either by directly affecting their gating mechanisms or by modifying the local electric field around the neuron (Goychuk, 2018; Wu et al., 2022).

MF-based techniques offer a non-invasive alternative to surgical interventions, reducing risks associated with neurosurgery and increasing accessibility for patients. Additionally, non-invasive methods are better suited for repeated or prolonged treatments, which are often necessary for managing chronic conditions (Deblieck et al., 2021; Sawada and Yamakage, 2024). The versatility of MFs allows for a range of therapeutic outcomes depending on stimulation parameters. For example, extremely low-frequency EMFs (ELF-EMFs) have been shown to inhibit neuronal activity, reducing seizure frequency in epilepsy (Wang et al., 2024). Conversely, high-frequency fields can enhance neuronal activity, improving symptoms in conditions such as depression by targeting hypoactive brain regions (George and Post, 2011; Lefaucheur et al., 2014).

### 3.1 Effects of magnetic fields on cellular functions

The interaction of electric, magnetic, and EMFs with biological cells and tissues has been extensively studied. Despite notable progress, the precise mechanisms by which MFs influence living organisms remain incompletely understood, highlighting the need for further research. MFs can interact directly with moving charges, such as ions and proteins, as well as with magnetic materials within tissues, through various physical mechanisms (Hou et al., 2010).

Viewing living cells as electrochemical systems with charged components provides a useful framework for understanding how static MFs and EMFs can influence cellular functions. According to the “window effect” theory, the biological effects of MFs are typically observed within specific frequency or intensity ranges, suggesting that cells respond to physical stimuli in a manner analogous to their response to chemical signals such as hormones (Alipour et al., 2022a; Hajipour Verdom et al., 2018; Latypova et al., 2024; Wu et al., 2022). This theory implies that MFs can act as cellular messengers, eliciting physiological responses such as changes in cell survival, proliferation, apoptosis, differentiation, gene expression, protein synthesis, enzymatic activity, and ion homeostasis (Alipour et al., 2022a; Barati et al., 2021; Hajipour et al., 2017; Hajipour Verdom et al., 2018; Kashani et al., 2023; Krylov and Osipova, 2023; Ouyang, 2019; Wasak et al., 2019). Additionally, MFs have been explored for therapeutic applications, including pain relief and the treatment of certain medical conditions (Paolucci et al., 2020).

The biological effects of MFs depend on several factors, including the type of MF, its strength, frequency, pattern, and duration of exposure, and the specific properties of the target tissue (Hajipour Verdom et al., 2018). MFs interact with metals, ions, and charged or magnetic particles in tissues, potentially increasing the concentration, activity, and lifespan of reactive species such as reactive oxygen species (ROS) and reactive nitrogen species (RNS) (Alipour et al., 2022a; Hajipour Verdom et al., 2018; Schuermann and Mevissen, 2021). For example, paramagnetic metals can participate in ROS production through Fenton or Haber-Weiss reactions, which, at elevated levels, may cause oxidative damage to proteins, nucleic acids, and lipids, leading to cell death (Hajipour Verdom et al., 2018; Shabanpour et al., 2024). However, ROS also play essential roles as signaling molecules, regulating metabolic pathways and the activity of transcription factors such as AP-1 and Nrf-2 (Alipour et al., 2022a; Priya Dharshini et al., 2020).

Moreover, direct interactions between MFs and biological tissues can be described using Maxwell’s equations, though different tissue components exhibit varying responses to MFs (Chi et al., 2020). In the case of ELF-EMFs, the photon energy is insufficient to directly break chemical bonds or damage DNA molecules. For example, at frequencies of 50–60 Hz, the photon energy is approximately 10–12 times smaller than the energy required to break the weakest chemical bonds (Panagopoulos et al., 2021). However, MFs can exert a Lorentz force on moving charged particles, potentially influencing electrochemical currents within cells, particularly in neurons (Yachi et al., 2022). Low-frequency EMFs may induce cellular resonance effects (Tian et al., 2023), while high-frequency MFs can enhance localized energy absorption, modulating neuronal excitability (Moliadze et al., 2010). This dual mechanism indecates the potential of combining photon energy and MF application for targeted neuromodulation.

### 3.2 Impact of magnetic fields on tissue and organ levels

MFs have demonstrated the capacity to modulate brain activity at both tissue and organ levels, offering promising therapeutic potential for neurological disorders. These effects span multiple scales, from molecular and cellular changes within individual neurons to broader, network-level alterations that influence entire brain regions (Premi et al., 2018).

At the tissue level, MFs can modulate the collective activity of neuronal populations. Techniques such as TMS have shown the ability to non-invasively induce electrochemical currents in specific brain regions, either enhancing or suppressing neural activity depending on the frequency and intensity of the magnetic pulses (Maniglia et al., 2019). For example, repetitive TMS (rTMS) has been shown to induce long-lasting changes in cortical excitability, a phenomenon linked to synaptic plasticity. High-frequency rTMS was applied to the motor cortex has improved motor function in stroke patients by facilitating synaptic connections within motor pathways (de Freitas Zanona et al., 2023; Ni et al., 2023). Conversely, low-frequency rTMS has been used to suppress hyperactive brain regions, such as in patients with tinnitus or depression, where it reduces symptoms by downregulating overactive cortical areas (Adu et al., 2022). These effects are attributed to the ability of MFs to influence the excitability of neuronal circuits, thereby altering information flow within the brain (Asao et al., 2022). By targeting specific regions, MF-based therapies can modulate the balance of excitatory and inhibitory signals, offering therapeutic benefits for a range of neurological and psychiatric disorders (Turner et al., 2014).

At the organ level, MFs can influence larger brain networks, which play a critical role in coordinating functions such as motor control, cognition, and emotion (Cameron et al., 2019). MFs have the potential to reset dysfunctional circuits or enhance communication between brain regions (Dufor et al., 2023), providing therapeutic benefits for conditions such as major depressive disorder (MDD). For instance, rTMS targeting the dorsolateral prefrontal cortex (DLPFC)-a key region in mood regulation-has been shown to enhance connectivity within mood-related networks, leading to sustained improvements in depressive symptoms (Lu et al., 2024; Vida et al., 2023).

Magnetothermal stimulation, which uses MNPs to generate localized heating, represents another innovative approach. This technique allows precise targeting of deep brain structures that are difficult to reach with conventional TMS (Fiocchi et al., 2022; Le et al., 2019). In animal models, magnetothermal stimulation of the hypothalamus has been shown to influence body temperature regulation and metabolic processes, demonstrating the potential of MFs to modulate organ-level functions beyond the brain itself (Jang et al., 2023).

The therapeutic potential of MFs at the tissue and organ levels has been explored in various neurological conditions:

1. Epilepsy: Low-frequency rTMS has been used to suppress epileptic activity by targeting seizure-prone brain regions. Studies have shown reductions in seizure frequency in patients with drug-resistant epilepsy, likely due to modulation of excitability in epileptogenic zones (Balestrini and Sander, 2020; de Labra et al., 2023).
2. Parkinson’s disease: rTMS applied to the motor cortex and basal ganglia has improved motor symptoms, including tremors, by modulating disrupted dopaminergic pathways (Spagnolo et al., 2021).
3. Chronic pain: rTMS targeting brain regions involved in pain processing, such as the primary motor cortex and anterior cingulate cortex, has reduced pain in conditions such as fibromyalgia and neuropathic pain. This effect is thought to result from alterations in the pain matrix within the brain (Ciampi de Andrade and García-Larrea, 2023; Pinot-Monange et al., 2019).

The ability of MFs to modulate brain activity at the tissue and organ levels has significant therapeutic implications. As research continues to elucidate the underlying mechanisms, there is potential for developing more effective and targeted treatments. Combination of MF-based therapies with other modalities, such as pharmacotherapy or behavioral interventions, could further enhance outcomes. Advances in neuroimaging and computational modeling are expected to improve the precision of brain network targeting, leading to more personalized neuromodulation strategies.

### 3.3 Current therapeutic applications of magnetic fields

MF-based neuromodulation techniques, particularly rTMS, have emerged as powerful tools for treating a range of neurological and psychiatric conditions. Supported by a growing body of research, these non-invasive methods modulate brain activity and have shown efficacy in managing disorders such as depression, schizophrenia, and multiple sclerosis (Alashwal et al., 2023).

rTMS uses rapidly changing MFs to induce electrochemical currents in specific brain regions, modulating neuronal activity through depolarization or hyperpolarization. Depending on the stimulation frequency, rTMS can either enhance or suppress neuronal excitability, making it a versatile tool for treating conditions such as depression, anxiety, and neuropathic pain (Lu et al., 2024).

rTMS involves applying repetitive magnetic pulses through a coil placed on the scalp, which induces electrochemical currents in underlying brain tissue (Tubbs and Vazquez, 2024). The effects of rTMS depend on the frequency, intensity, and duration of stimulation, as well as the specific brain region being exposed (Han et al., 2023). Coil designs, such as figure-of-eight or H-coils, are engineered to optimize MFs strength and focality, ensuring precise stimulation of the desired neural circuits (Deng et al., 2014). Additionally, different pulse types-such as monophasic, biphasic, and burst stimulation-enable frequency-dependent modulation of neuronal activity (Wendt et al., 2023). Stimulation protocols, including conventional rTMS and theta-burst stimulation (TBS), are customized to achieve specific therapeutic outcomes (Liu et al., 2024).

One of the most well-established and clinically validated applications of rTMS is in the treatment of MDD, particularly for patients who have not responded to conventional therapies such as antidepressant medications or psychotherapy (Lu et al., 2024). The non-invasive nature of rTMS, coupled with its relatively mild and transient side effects-such as scalp discomfort or mild headaches-makes it a compelling alternative for individuals with treatment-resistant depression (Lanza et al., 2023). Standard rTMS therapy for MDD typically involves daily sessions administered over a period of four to six weeks, with each session lasting approximately 30 to 40 min (Al-Ruhaili et al., 2023). This structured regimen has been shown to significantly improve depressive symptoms in many patients, offering hope for those who have struggled to find relief through other treatment modalities.

rTMS has also shown promise in treating bipolar disorder, particularly for depressive episodes. Preliminary studies suggest it could serve as an effective adjunctive therapy, though further research is needed to establish its efficacy (Nguyen et al., 2021). In schizophrenia, rTMS has been used to target the temporoparietal cortex, a region associated with auditory hallucinations. Low-frequency rTMS (1 Hz) applied to this area has reduced the frequency and severity of hallucinations in some patients (van Lutterveld et al., 2012). Additionally, rTMS targeting the prefrontal cortex has been explored for improving cognitive function and alleviating negative symptoms such as social withdrawal and apathy (Kos et al., 2024). While not yet a first-line treatment, rTMS is increasingly used as an adjunctive therapy for medication-resistant symptoms (Wagner et al., 2021; Xie et al., 2023).

rTMS has been investigated for alleviating symptoms of multiple sclerosis (MS), a neurodegenerative disorder characterized by motor dysfunction, fatigue, and cognitive impairment (Ahmadpanah et al., 2023). High-frequency rTMS targeting the motor cortex has shown potential in improving motor performance and reducing spasticity by enhancing cortical excitability and promoting neuroplasticity (Hassan et al., 2021). Studies have also explored its use for fatigue and cognitive symptoms, though results have been mixed (Gaede et al., 2017; Iglesias, 2020). Despite variability in outcomes, rTMS offers a non-invasive and well-tolerated option for managing certain MS symptoms, particularly in patients unresponsive to conventional therapies.

Beyond rTMS, other MF-based techniques are being explored for their therapeutic potential:

1. tDCS: a non-invasive technique that applies weak electrical currents to the scalp to modulate cortical activity. tDCS has been studied for conditions such as depression, chronic pain, and cognitive impairments. While less established than rTMS, it offers a more portable and cost-effective alternative (Klírová et al., 2024).
2. DBS: an invasive surgical procedure that involves implanting electrodes into specific regions of the brain (Krauss et al., 2021). It has demonstrated remarkable efficacy in treating movement disorders, such as Parkinson’s disease and dystonia. However, its application in chronic pain management is less established and warrants further research to fully understand its potential benefits (Krauss et al., 2021). Despite being a surgical intervention, DBS highlights the broader promise of MF-based technologies in the field of neuromodulation, offering insights into non-invasive alternatives for targeted brain stimulation.

Unlike DBS, which is a highly targeted, tCS provides non-specific stimulation across cortical areas. This distinction highlights the need for advanced techniques such as magnetothermal stimulation, which combines the precision of targeted interventions with the non-invasive nature of methods such as tCS (Zhang E. et al., 2024).

While MF-based neuromodulation holds great promise, several challenges remain. Precise targeting of ion channels without affecting surrounding tissues requires further technological refinement. Additionally, the long-term effects of MF exposure on brain function and structure are not yet fully understood, necessitating comprehensive safety studies (Borrione et al., 2020; Singh et al., 2020).

Standard non-invasive techniques such as tDCS and TMS are limited in their ability to reach deep brain regions effectively (Luigjes et al., 2019). These methods typically influence on superficial cortical areas, making it challenging to modulate deeper neural circuits that are critical in conditions such as epilepsy, Parkinson’s disease, and treatment-resistant depression. Magnetothermal stimulation overcomes this challenge by using MFs and nanoparticles to enable precise and targeted neuromodulation deep within the brain (Yuan et al., 2022). This innovative approach not only expands the range of treatable neurological and psychiatric conditions but also holds the potential to enhance therapeutic outcomes significantly.

As research progresses, there is growing interest in personalized approaches to neuromodulation. By tailoring stimulation parameters to individual neurophysiological profiles, clinicians can optimize treatment efficacy and patient outcomes. Furthermore, combining MF-based therapies with complementary modalities, such as pharmacotherapy or behavioral interventions, may further enhance therapeutic results.

## 4 Magnetothermal neurostimulation

Magnetothermal stimulation is an innovative neuromodulation technique that utilizes the interaction between MFs and functionalized nanoparticles to achieve precise, non-invasive control of neural activity. A key advantage of this approach is its ability to remotely and selectively modulate specific brain regions, providing a highly targeted alternative to invasive procedures. This method holds significant promise for treating neurological disorders associated with abnormal neural activity, including epilepsy, chronic pain, and mood disorders (Hescham et al., 2021; Sheth and Mayberg, 2023).

**FIGURE 2:**
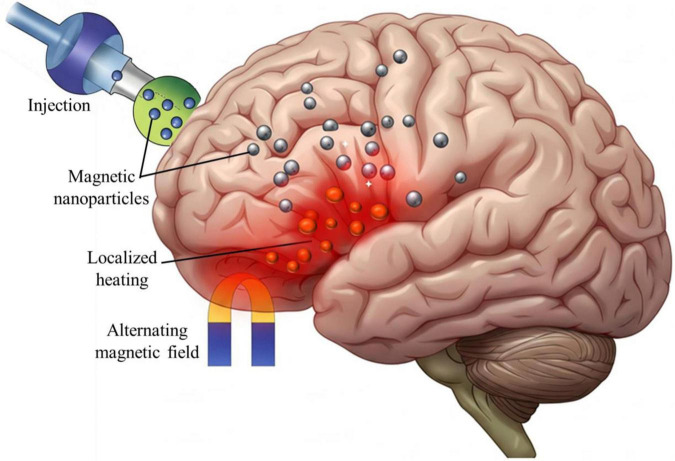
The schematic of magnetothermal stimulation process. Designed magnetic nanoparticles (MNPs) are targeted and accumulate in the specific regions of brain, where an external alternating magnetic field (AMF) induces localized heating (highlighted in red). This controlled thermal energy modulates ion channel activity and neuronal excitability, offering a promising non-invasive strategy for neuromodulation therapies.

Magnetothermal stimulation relies on the application of alternating magnetic fields (AMFs) to heat functionalized MNPs localized in targeted brain regions (Figure 2). This localized heating modulates ion channel activity and neuronal excitability, enabling precise control over neural circuits (Munshi et al., 2017).

A notable advantage of this technique is its ability to achieve deep brain modulation without the need for direct physical access. By functionalizing MNPs with specific ligands, researchers can enhance their specificity for targeted neural circuits, further improving the precision of the intervention (Dash et al., 2022). This level of control is particularly valuable for treating conditions where abnormal activity is localized to specific brain regions.

While magnetothermal stimulation and focused ultrasound (FUS) both offer non-invasive targeting of deep brain regions, they differ in their mechanisms and applications. FUS uses acoustic energy to influence neuronal activity, eliminating the need for nanoparticles and simplifying its application (Jiao et al., 2024). However, FUS lacks the inherent ability to functionalize targeting agents, which limits its specificity compared to magnetothermal stimulation. Additionally, FUS requires careful calibration to avoid unintended tissue heating or cavitation, which can pose risks during prolonged use (Kim et al., 2014).

Preclinical studies have demonstrated the efficacy and potential of magnetothermal stimulation in modulating neural activity and treating neurological disorders. For example, Munshi et al. (2017) used functionalized MNPs to selectively stimulate the motor cortex in a rodent model. By applying AMFs at 500 kHz, the study achieved precise modulation of neuronal activity, as evidenced by changes in motor behavior and electrophysiological recordings (Munshi et al., 2017).

Another study by Hescham et al. (2021) explored the use of magnetothermal stimulation to alleviate chronic pain in a mouse model. The researchers coated MNPs with ligands targeting pain-related neural circuits and applied AMFs at 160 kHz. The results showed significant reductions in pain behaviors, with minimal off-target effects, underscoring the potential of magnetothermal stimulation for non-invasive pain management (Hescham et al., 2021).

The integration of magnetothermal stimulation with other neuromodulation methods, such as FUS or pharmacological therapies, could expand its therapeutic applications. Furthermore, combining this approach with advanced imaging techniques may enable real-time monitoring, enhancing the precision, efficacy, and safety of neural modulation.

### 4.1 Magnetic nanoparticles for neuromodulation

MNPs represent a cutting-edge approach in neuromodulation, offering a highly targeted method to influence neuronal ion channels and modulate brain activity. These nanoparticles can be introduced into the body and precisely directed to specific brain regions using external MFs (Latypova et al., 2024; Signorelli et al., 2022). The physical mechanisms of MNPs in neuromodulation can be broadly categorized into three hypotheses:

1. Localized heating: AMFs induce localized heating of MNPs through Neel and Brownian relaxation processes. This thermal fluctuations can influence the kinetics and gating behavior of temperature-sensitive ion channels, modulating neuronal activity (Huang et al., 2023; Leuchtag, 2023).
2. Mechanical effects: MFs generate torque or force on MNPs, causing mechanical stress on neuronal membranes or ion channels. This stress can open mechanically-gated ion channels or alter membrane properties, influencing excitability (Barbic, 2019; Gribanovsky et al., 2022).
3. Direct electromagnetic interaction: MNPs generate localized EMFs that interact with neuronal structures, influencing ion flow through electromagnetic induction or modulating membrane potentials (Liu N. et al., 2022; Yu et al., 2022).

While these hypotheses provide a framework for understanding MNP-based neuromodulation, several challenges remain. For instance, the extent of localized heating and its specificity to target neurons need further investigation (Roet et al., 2019). Similarly, the magnitude of mechanical effects and their impact on neuronal membranes require quantification to establish thresholds for therapeutic efficacy (Romero et al., 2022). Moreover, advances in imaging and simulation techniques are essential to bridge these gaps and validate the proposed mechanisms *in vivo* (Ge et al., 2024). To overcome these challenges, researchers are exploring the use of high-resolution imaging modalities, such as MRI and optogenetic tools, to visualize MNP behavior in real-time. Additionally, computational models that simulate MNP-neuron interactions under various field conditions can provide insights into optimizing neuromodulation protocols (Alipour and Zali, 2022; Alipour et al., 2022b; Kamkar et al., 2022).

One of the most promising aspects of MNPs is their ability to achieve precise targeting of specific brain regions. By functionalizing the surface of MNPs with ligands, antibodies, or peptides, these particles can be engineered to selectively bind to specific neuronal populations or ion channels. This specificity allows for the modulation of distinct neural circuits, offering highly selective therapeutic interventions (Dominguez-Paredes et al., 2021; Liu et al., 2020).

For example, MNPs can be directed to regions such as the basal ganglia in Parkinson’s disease, where they modulate voltage-gated ion channels in affected neurons, potentially restoring normal function and alleviating symptoms (Xu et al., 2023). Delivery methods for MNPs include systemic administration (e.g., intravenous injection) or localized application (e.g., intracranial infusion), with external MFs guiding the nanoparticles to the desired site to enhance precision and minimize off-target effects (Ashoori et al., 2023; Kazemi-Ashtiyani et al., 2022; Satari et al., 2024).

The interaction of MNPs with ion channels is grounded in neurobiophysical principles. Localized heating alters ion permeability coefficients, as described by the Goldman-Hodgkin-Katz (GHK) equation (Equation 1), influencing membrane potential (Vm) and neuronal excitability. Changes in membrane conductance (gm) and equilibrium potential (Em) can be quantified using Ohm’s law, illustrating the direct impact of magnetothermal stimulation on neural activity (Alvarez and Latorre, 2017).

The thermal energy generated by MNPs can also influence the surrounding cellular environment, affecting membrane fluidity, enzyme activity, and metabolic processes. These indirect effects further modulate ion channel function and neuronal signaling, demonstrating the multifaceted nature of MNP-based neuromodulation (Evtushenko et al., 2023; Li et al., 2021; Montalbetti et al., 2021).

Preclinical studies have demonstrated the promising potential of MNPs for neuromodulation in animal models. For example, MNPs have been successfully used to modulate activity in the motor cortex of rodents, resulting in changes in motor behavior and electrophysiological responses (Hescham et al., 2021; Romero et al., 2022; Wu et al., 2021). Similarly, MNPs targeting circuits associated with pain have shown efficacy in alleviating chronic pain in mouse models, with minimal off-target effects (Wu et al., 2017).

A key advantage of MNPs is their capacity for repeated and adjustable interventions. The same population of nanoparticles can be activated multiple times through the reapplication of a MF, offering a flexible, reversible, and non-invasive approach to neuromodulation (Roet et al., 2019). However, the clinical translation of MNPs for magnetothermal stimulation in humans faces several safety challenges that must be addressed:

1. Toxicity of MNP materials: Some nanoparticle coatings or core materials may trigger cytotoxicity or inflammatory responses. This risk can be mitigated by using biocompatible coatings such as polyethylene glycol (PEG) or silica, which reduce immune recognition and enhance biostability (Park et al., 2023). Additionally, ROS generated during magnetothermal stimulation play a dual role in neuromodulation. While moderate ROS levels can act as signaling molecules, influencing neuronal plasticity and synaptic function, excessive ROS production must be carefully controlled to prevent oxidative stress and cellular damage (Tommasini et al., 2024).
2. Long-term retention: The accumulation of MNPs in tissues raises concerns about chronic inflammation or interference with normal physiological processes. Strategies such as designing biodegradable nanoparticles or developing techniques for efficient clearance from the body are critical (Ansari et al., 2024).
3. Localized heating risks: Overheating during magnetothermal stimulation can cause damage to surrounding tissues. Ensuring safety during therapy requires real-time temperature monitoring and precise control of MF parameters (Shoshiashvili et al., 2023).
4. Off-target effects: Non-specific accumulation of MNPs in unintended tissues can reduce therapeutic efficacy and lead to unintended side effects. Functionalization of nanoparticles with specific ligands can improve targeting precision and minimize off-target effects (Aram et al., 2022; Markides et al., 2012).

To overcome these challenges, future studies should focus on developing advanced imaging techniques, such as MRI, to track the real time biodistribution of MNPs (Ebrahimpour et al., 2022; Remmo et al., 2024). Computational modeling of MF interactions with biological tissues can also help optimize parameters to minimize risks (Singh et al., 2024). Collaboration between materials scientists, biophysicists, and clinicians will be essential to design and test biocompatible MNPs that meet regulatory standards for human use.

Furthermore, optimizing the size, composition, and surface chemistry of MNPs is essential for maximizing their efficacy while minimizing adverse effects (Dehghani et al., 2024; Shamsipur et al., 2024). Advance in nanotechnology and materials science are expected to drive the development of more sophisticated MNPs capable of precise and controlled modulation of ion channels. These innovations could pave the way for novel treatments for a range of neurological disorders, including chronic pain, epilepsy, and neurodegenerative diseases.

### 4.2 How magnetothermal stimulation works

Magnetothermal stimulation uses the unique properties of MNPs to achieve precise and localized neuromodulation. This technique is based on the ability of MNPs to convert energy from an AMF into heat (Castillo-Torres and Páez-Maggio, 2022; Chen et al., 2015). The amount of heat generated depends on several factors, including the magnetic susceptibility of the nanoparticles, their size, the strength of the applied MF, and the frequency of the AMF (Kuppe et al., 2020; Ovejero et al., 2021). Typically, magnetothermal stimulation utilizes AMFs with field strengths ranging of 10–100 mT and frequencies between 100 kHz and 1 MHz (Kim et al., 2024; Kuckla et al., 2023). These parameters are carefully optimized to ensure efficient energy transfer to the MNPs while minimizing off-target heating and potential tissue damage. The MFs are generated using specialized devices such as Helmholtz coils or solenoids, which create uniform AMFs around the target region. For clinical applications, wearable or implantable MF generators are being developed to facilitate localized and patient-specific neuromodulation (Davidson et al., 2024; Oh et al., 2024; Sarica et al., 2021).

Generally, magnetothermal stimulation offers several advantages over established MFs-based neuromodulation methods:

1. Precision and localization: By combining MFs with functionalized MNPs, this approach enables highly localized modulation of neuronal activity (Ko et al., 2022). The heat generated is confined to the region containing the nanoparticles, minimizing effects on surrounding tissues.
2. Targeting specificity: Functionalized MNPs can be engineered to bind to specific neuronal populations or ion channels, enhancing the precision of neuromodulation (Zhang et al., 2023). This allows for targeted modulation of deep brain regions or specific neural circuits that are difficult to reach with conventional techniques.
3. Non-invasiveness: Unlike invasive methods such as electrical DBS, magnetothermal stimulation does not require surgical intervention, reducing risks and improving patient accessibility (Hescham et al., 2021).
4. Controlled modulation: The ability to adjust MF parameters (strength, frequency, and duration) allows for modified control over the thermal effect, enabling personalized therapeutic interventions (Chang et al., 2025).

### 4.3 Advantages of remote neuromodulation

The development of remote neuromodulation techniques, particularly those utilizing MFs, represents a transformative advancement in neuroscience. These methods combine precision, safety, and versatility, offering significant advantages for both research and clinical applications (Zhi et al., 2025). By enabling non-invasive modulation of neuronal activity, remote neuromodulation introduces a new paradigm in the treatment of neurological and psychiatric disorders.

One of the most compelling advantages of remote neuromodulation is its non-invasive nature. Techniques such as DBS require surgical implantation of electrodes, which carries risks such as infection, bleeding, and complications from anesthesia (Shoshiashvili et al., 2023). In contrast, MF-based methods, such as TMS and magnetothermal stimulation, modulate brain activity without penetrating the skull or brain tissue. This eliminates the need for surgery, reducing the risk of adverse effects and avoiding the recovery time associated with invasive procedures (Radyte et al., 2022). Indeed, remote neuromodulation offers a high degree of precision in targeting specific neuronal circuits. This precision is achieved by carefully calibrating MF parameters, such as strength, frequency, and focus, allowing for the modulation of neural pathways implicated in various (Park et al., 2020; Zhang et al., 2023).

The non-invasive nature of these techniques enhances the accessibility of neuromodulation to a broader range of patients, including those who are ineligible for surgery due to underlying health conditions. Furthermore, the ability to administer these treatments repeatedly without surgical intervention makes them ideal for managing chronic conditions that require ongoing therapy (Lv et al., 2023). This approach is especially beneficial for populations traditionally underserved by surgical interventions, such as pediatric patients, the elderly, and individuals with comorbidities that increase surgical risks (Ege et al., 2023). Additionally, the reversibility of remote neuromodulation allows treatments to be adjusted or discontinued without the long-term consequences associated with implanted devices, expanding therapeutic possibilities without the need for invasive procedures (Zanos, 2019).

The use of functionalized MNPs further enhances specificity, as they can be engineered to bind selectively to specific neuron types or ion channels, enabling highly targeted neuromodulation. This precision is particularly beneficial for treating conditions such as epilepsy, chronic pain, and depression, where dysregulation neural circuits plays a key role (Yu et al., 2021). Additionally, the ability to modify treatments based on patient response improves therapeutic outcomes and minimizes side effects. This flexibility supports the advancement of precision medicine, where treatments are customized to the unique characteristics of each patient.

As remote neuromodulation technologies continue to advance, their potential applications are poised to expand significantly. Innovations in imaging, computational modeling, and nanotechnology will further refine the precision and efficacy of these techniques. For example, integrating real-time neuroimaging with MF-based neuromodulation could allow for dynamic treatment adjustments, optimizing outcomes for patients with complex neurological conditions.

## 5 Conclusion

The MF-based neuromodulation, particularly magnetothermal stimulation presents a promising, non-invasive approach for modulating neuronal activity. However, its translation into clinical practice faces significant technical and biological challenges. A primary hurdle is achieving precise targeting of specific ion channels within neuronal populations without affecting surrounding tissues. Ongoing research focuses on developing functionalized nanoparticles and advanced imaging technologies to enhance targeting accuracy and minimize off-target effects. Additionally, the long-term safety of magnetic exposure remains a critical concern, as its impact on neural plasticity and overall brain function is not yet fully understood. Addressing these issues will require extensive animal studies and clinical trials to ensure both efficacy and safety.

The potential clinical applications of magnetothermal neuromodulation are extensive, offering hope for non-invasive treatments for a variety of neurological and psychiatric disorders. For example, it could provide alternatives to surgical interventions in epilepsy, modulate dysregulated pain networks in chronic pain conditions, and offer therapeutic benefits for neurodegenerative diseases such as Alzheimer’s. In psychiatry, this technology holds promise for treating treatment-resistant depression, anxiety disorders, and schizophrenia by targeting specific neural circuits. Beyond therapeutic applications, magnetothermal neuromodulation could play a significant role in cognitive enhancement and rehabilitation, aiding in stroke recovery, traumatic brain injury treatment, and maintaining cognitive function in older adults.

The future of this field lies in personalized medicine, where precision-targeted and adaptive neuromodulation protocols are customized to individual patient responses. By integrating real-time imaging, computational modeling, and artificial intelligence, researchers can optimize treatment parameters and improve therapeutic outcomes. This manuscript synthesizes current advancements in MNP-based neuromodulation, examining its underlying mechanisms-such as localized heating, force generation, and electromagnetic induction-while highlighting its potential applications. Although the field continues to evolve, this review emphasizes the importance of bridging theoretical insights with practical applications to develop more precise and effective neuromodulation therapies.

To fully realize the potential of magnetothermal neuromodulation, future research should focus more deeply on the fundamental biophysical interactions between MFs and biological tissues, particularly the effects of MFs on ion channels and cellular pathways. Innovations in hardware, nanoparticle engineering, and the integration of real-time imaging and artificial intelligence will enhance the precision and effectiveness of these therapies. Comprehensive studies are essential to evaluating the long-term effects of MF exposure, ensuring both safety and therapeutic efficacy. Expanding research into new conditions, combination therapies, and preventative care strategies will further broaden the scope of magnetothermal neuromodulation. As the technology advances, addressing ethical concerns-such as informed consent, privacy, and societal implications-will be crucial for its responsible development and implementation.

In summary, magnetothermal neuromodulation represents a transformative approach to treating neurological and psychiatric disorders, with the potential to revolutionize personalized medicine. By addressing current challenges and advancing research in key areas, this technology could provide new hope for patients and significantly improve clinical outcomes.
